# Macrophage Heterogeneity in Liver Ischemia-Reperfusion Injury

**DOI:** 10.21203/rs.3.rs-8347196/v1

**Published:** 2025-12-22

**Authors:** Yuan Zhai, Yue Wang, Cheng Zhong, Jackson Chin, Nan Xia, Yi-Chu Kao, Tori Huey, Hesham El-Shewy, Dirk Van der Windt, Kris Helke, Thomas Morinelli, Aaron Meyer

**Affiliations:** College of Medicine, Medical University of South Carolina; Medical University of South Carolina; Medical University of South Carolina; University of california-Los Angeles; Medical University of South Carolina; Medical University of South Carolina; Medical University of South Carolina; Medical University of South Carolina; Medical University of South Carolina; Medical University of South Carolina; Medical University of South Carolina; University of California, Los Angeles

**Keywords:** Kupffer cells, infiltrating macrophages, IL-1a, IL-1b, Trem2

## Abstract

Kupffer cells (KCs) are implicated in liver ischemia reperfusion injury (IRI). However, their precise roles vs. bone marrow–derived infiltrating macrophages (iMФs) remain controversial. In this study, we used Clec4F-tdTomato (DTR) mice to track KC-specific changes and assessed their function by DT-mediated depletion in the acute phase of liver IRI. We found that liver IR leads to substantial loss of embryonically derived Clec4F^+^TIM-4^+^ KCs, coinciding with pronounced infiltration of monocytes and neutrophils. A single dose of DT resulted in a complete replacement of Clec4F^+^ KCs with iMФs, leading to a temporally dynamic modulation of liver susceptibility to IRI: reduced at 24 hours but aggravated at 14 days following DT administration. The early liver protection was mediated by Gr-1^+^CD11b^+^ iMФs with high expression of Trem2. Anti-Gr-1 Abs or Trem-2 blockade abolished the cytoprotective phenotype and restored liver IRI. Differential gene expression analysis between KCs and iMФs revealed that KC Inflammatory responses were driven primarily by IL-1 family genes. KC replacement with iMФs resulted in significantly lower levels of IL-1a and IL-1b, gene expression in both sham and IR livers. Ab-mediated neutralization of either of these cytokines reduced liver IRI in KC-dependent manner. Together, these findings identify Gr-1^+^Trem-2^+^ iMФs as immunoregulatory players that protect livers from IRI and highlight an IL-1a/b dependent pro-inflammatory role of KCs.

## Introduction

Ischemia-reperfusion injury (IRI) is an unavoidable pathological consequence of both surgical and non-surgical liver conditions, including hepatic tumor resection, transplantation, and trauma (1–3). The pathogenesis of liver IRI is primarily driven by innate immune-mediated inflammation (4–7). Among innate immune cells, macrophages are the most abundant in the liver and play pivotal roles in orchestrating the inflammatory response to IRI. Liver macrophages are heterogeneous (8–10). Kupffer cells (KCs), the largest population of tissue-resident macrophages in the body, originate from embryonic precursors distinct from bone marrow-derived monocytes. During liver injury or infection, circulating monocytes are recruited and infiltrate the liver, differentiating into infiltrating macrophages (iMФs). Although both KCs and iMФs have been implicated in liver IRI (5, 7), the potential distinctive roles remain unclear, partly due to the lack of lineage-tracing and cell-specific targeting tools.

Previous studies have employed CD11b-DTR, CCR2 knockout (KO) mice, and clodronate liposomes (CLs) to study macrophage involvement in liver IRI (11–13). However, these approaches are limited: KCs express low/no levels of CD11b and CCR2, and CLs exhibit off-target effects and systemic toxicity. Our work has demonstrated that liver immune activation during IR occurs in two phases. In addition to the infiltration of peripheral Gr1^+^ myeloid cells, necrotic depletion of KCs itself acts as a pro-inflammatory trigger. We previously showed that the necroptosis inhibitor Nec-1s mitigates KC loss and protects the liver from IRI (14). More recently, we used anti-TIM-4 antibodies to selectively target tissue-resident macrophages (15). Interestingly, we observed a time-dependent dichotomy in the antibody’s effects—either attenuating or exacerbating liver IRI—likely due to the kinetics of macrophage repopulation following depletion. KC loss rapidly induces monocyte infiltration, with subsequent differentiation and reprogramming in the liver. Therefore, the extent of KC replacement by iMФs and their functional state may critically influence liver susceptibility to IRI. However, since TIM-4 is a general marker of tissue-resident macrophages and the mechanism of anti-TIM-4 action (depletion vs. downregulation) remains debated, more specific tools are needed to clarify the role of KCs in liver IR.

Recent RNA-seq analyses have identified Clec4F as the most specific marker for murine KCs (8, 10, 16, 17). The development of Clec4F-tdTomato and Clec4F-DTR mice enables us to precisely target KCs in vivo (16, 17). In this study, we investigated how liver IR affects KCs and how KC depletion and replacement influence the pathophysiology of liver IRI. For comparison, we also utilized CD11b-DTR and CCR2-GFP mice to assess the role of bone marrow-derived MФs. Our findings reveal that KCs are critical determinants of liver susceptibility to IRI and they promote liver inflammatory injury by producing IL-1a/b. In contrast, broad inhibition of monocyte/macrophage infiltration has minimal protective effect. A subset of Gr-1^+^CCR2^+^Trem2^high^ monocytes in circulation exhibits immune-regulatory properties and confers protection against liver IRI.

## Materials and Methods

### Sex as a biological variable.

Both male and female mice are used in our experiments. Female mice generally develop less severe liver IRI under the same experimental condition, as compared with their male counterparts. We chose to use data with male cohorts, unless specified.

### Animals.

Clec4F-cre, iDTR, CD11b-DTR, CCR2-GFP and WT C57BL/6 mice (6–8 weeks old) were purchased from the Jackson Laboratory (Bar Harbor, ME), and housed in the Medical University of South Carolina (MUSC) animal facility under specific pathogen-free conditions, and received humane care according to the criteria outlined in the “Guide for the Care and Use of Laboratory Animals” prepared by the National Academy of Sciences and published by the National Institute of Health. All animal experiments were approved by MUSC Office of Animal Research Oversight Committee.

### Mouse liver partial warm ischemia model.

After a midline laparotomy, mice were injected with heparin (100 mg/kg) and an atraumatic clip was used to interrupt arterial/portal venous blood supply to the cephalad-liver lobes. After 90 or 60 min of ischemia, the clip was removed initiating hepatic reperfusion. Sham controls underwent the same procedure, but without vascular occlusion. Mice were sacrificed after 6h (peak of acute liver injury) or 24h to 7 days of reperfusion, and liver and serum samples were collected. Serum alanine aminotransferase (sALT) levels were measured using a colorimetric activity assay kit (Cayman, Ann Arbor MI). Part of liver specimens was fixed in 10% buffered formalin and embedded in paraffin. Liver sections (4mm) were stained with hematoxylin and eosin (HE). The severity of liver IRI was graded blindly using Suzuki’s criteria on a scale from 0 to 4. No necrosis, congestion/centrilobular ballooning is given a score of 0, while severe congestion and > 60% lobular necrosis is given a score of 4.

Antibodies including anti-Trem2 (Clone 178), anti-Gr-1(Clone RB6–8C5), anti-IL-1a (Clone ALF-161) and IL-IL-1b (Clone B122) were purchased from BioXcell (Lebanon, NH). Abs were administered i.v. at 0.25–0.5mg/mouse at either 24h or 1h prior to the onset of liver ischemia.

### Liver NPC/Kupffer cell isolation.

Liver NPC/KCs were isolated from normal or IR livers of B6 mice by in situ collagenase perfusion. In brief, livers were perfused via the portal vein with calcium- and magnesium-free HBSS supplemented with 2% heat-inactivated FBS, followed by 0.27% collagenase IV (Sigma, St Louis, MO). Perfused livers were dissected and teased through 70 mm nylon mesh cell strainers (BD Biosciences, San Diego, CA). Non-parenchymal cells (NPCs) were separated from hepatocytes by centrifuging at 50g for 2 min three times. NPCs were stained with fluorescence-labeled Abs and analyzed by FACS.

### Flow Cytometry.

Liver non-parenchymal cells (NPCs) were isolated from sham or IR livers, as described above. 1×10^6^cells were incubated first with rat anti-mouse CD16/32 for 10 minutes, followed by staining with fluorochrome-labeled antibodies: CD45, CD64, F4/80, CD11b, Gr1 (Clone: RB6–8C5), TIM-4, Ly6C, Ly6G, Trem2, or isotype-matched control antibody (eBioscience, San Diego, CA) for 20 minutes. Cells were washed with PBS and subjected to flow cytometry analysis with FACSCalibur (BD Biosciences).

### Macrophage Depletion.

To deplete KCs or CD11b + infiltrating macrophages, we injected diphtheria toxin at 10mg/g mouse body weight, i.v. 24h prior to the onset of liver ischemia. The selective depletion of KCs and CD11b macrophages in these mouse models as confirmed by FACS analysis of liver non-parenchymal cells and peritoneal exudes 24h post DT injection. As shown in Sup Fig. 1, KCs (F4/80 + Clec4F + TIM4+) were depleted in Clec4F-DTR, but not CD11b-DTR mice, while peritoneal macrophages (F4/80 + CD11b+) were depleted in CD11b-DTR, but not Clect4F-DTR mice.

### Quantitative RT-PCR.

Total RNA (2.5μg) was reverse-transcribed into cDNA using SuperScriptTM III First-Strand Synthesis System (Invitrogen, Carlsbad, CA). Quantitative-PCR was performed using the Azure Cielo (Azure Biosystems, Dublin, CA). In a final reaction volume of 20 μl, the following were added: 1x SuperMix (PowerTrackTM SYBR Green Master Mix Kit, Applied Biosystems, Foster City, CA), cDNA and 0.5 mM of each primer. Amplification conditions were: 95oC (2 min) followed by 40 cycles of 95 oC (15 s), 60 oC (30 s). The primers for mouse gene fragments, including TNF-a, IL-10, are listed in Table 1.

### RNA-seq analysis of KC and 24h iMФs.

Measurements for KCs and iMФs 24 hours post-depletion were collected from NCBI’s Gene Expression Omnibus under accession number GSE128657. Genes with a maximum read count below 10 across samples were removed; ribosomal genes (prefixed with “RPL” or “RPS”) and spliceosome genes (prefixed with “SNP” or “SNR”) were removed. Average gene expression was compared between KCs and 24h iMФs via two-sample t-test; resulting p-values were corrected for multiple-hypothesis testing via Benjamini-Hochberg procedure. Concurrently, log_2_-fold change in average gene expression between KCs and 24h iMФs was derived. Genes with (1) a log_2_-fold change greater than 1 and (2) a corrected t-test p-value less than 0.05 were considered differentially expressed (DEGs). Gene set enrichment analyses were performed via Enrichr through the GSEApy package to identify concerted biological trends in DEGs (18, 19). DEGs were compared against the Gene Ontology: Biological Process 2025 terms (20, 21).

### Computational Methods.

Computational analyses were performed with Python 3.11. Two-sample, independent t-tests were performed via SciPy (22). The Benjamini-Hochberg procedure for multiple hypotheses testing was implemented via statsmodels (23). Enrichment analyses were performed through the GSEApy package (18).

### Statistical analysis.

Results are shown as mean ± SD. Statistical analyses were performed using unpaired Student’s t-test with p<0.05 (two-tailed) considered as significant.

## Results

### 1. Liver IR results in significant depletion of embryonic KCs.

In the murine liver partial warm ischemia model (24), we have previously reported the depletion of KCs after liver ischemia reperfusion (14). As F4/80 and CD11b were common markers of MФ, which could not differentiate KC and iMФs, we took advantage of the KC-specific marker Clec4F to re-analyze KC response in liver IRI. In Clec4F-driven tdTomato reporter mice, we isolated non-parenchymal cells (NPCs) from sham or IR (60m or 90m) livers (both ischemia and non-ischemic lobes) at 24h post reperfusion.

**Figure 1:**
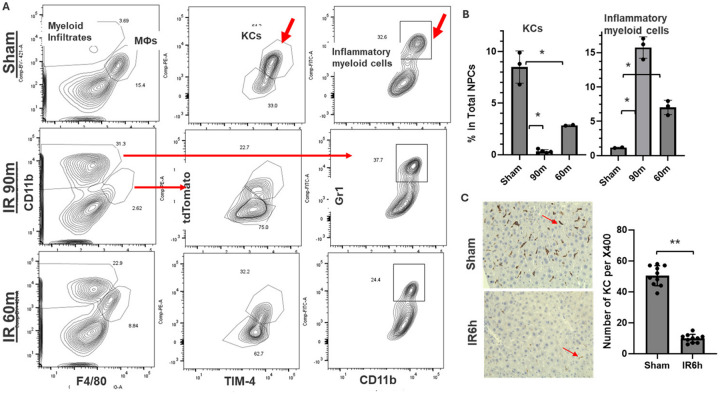
Liver IR depletes resident KCs. Liver NPCs were isolated from sham, 90m or 60m ischemia liver lobes of Clec4F-tdTomato reporter mice at 24h post reperfusion. Cells were stained with fluorochrome-labeled Abs and analyzed by FACS. (A) Representative plots of different experimental groups: NPCs were first plotted based on F4/80 and CD11b. MФs were further analyzed for Clec4F(tdTomato) and TIM-4. Myeloid infiltrates were further plotted based on CD11b and Gr-1. (B) Plots of average % of resident KCs (F4/80^+^CD11b^low^tdTomato^+^TIM-4^+^) and inflammatory myeloid cells (CD11b^high^Gr-1^+^) in total liver NPCs of different exp. groups. (C) Clec4F immunohistochemical staining of liver sections from sham and IR mice. Average numbers of Clec4F+ cells/microscopic area were plotted.

Resident KCs were identified as F4/80^+^CD11b^low^tdTomato^+^TIM-4^+^ cells. Compared with those in the sham livers (calculated based on the total# of cells isolated from IR livers and percentages of KCs), KCs were significantly depleted by liver IR: 90m ischemia resulted in more than 95% reduction, while 60m ischemia resulted in ~ 70% reduction, with simultaneous increases of infiltrating inflammatory myeloid cells (CD11b^high^Gr-1^+^) at 1500% and 500%, respectively (Fig. 1A, B). KCs in non-ischemic lobes were also affected with significant but less degree of reduction: ~55% reduction after 90m ischemia. Systemic inflammation (induced by LPS injection i.v.) also reduced KCs in livers (Supp Fig. 2). We confirmed KC depletion in IR livers by Clec4F immunohistochemical staining of liver sections. Indeed, average numbers of Clec4F + cells/area were significantly lower in IR livers vs. sham control (Fig. 1C).

In clinical liver transplantation, KC-specific markers have not been used to differentiate resident vs. infiltrating (or donor vs. recipient) macrophages. Based on CD14 and CD68 levels, it was shown that % KCs (CD45 + CD14 + CD68+) were indeed reduced with simultaneous increases of monocytes/macrophages (HLA-DR + CD14+) in liver allografts early after transplantation (25, 26). However, the magnitude of KC reduction was relatively small. As the duration of warm ischemia and time of biopsies post Tx (reperfusion) are both much shorter in human liver Tx, compared with our mouse model, we tested the impact of these two parameters on KC depletion. Indeed, KC depletion at 0h post reperfusion after 60min ischemia was not significant (based on % of KCs in total liver CD45 + cells vs. sham, Supplement Fig. 3). The depletion was significant at 1h post reperfusion (~ 50%), and a shorter ischemia time at 30m resulted in similar degree of KC depletion after 1h reperfusion time. Thus, KC depletion in clinical liver Tx biopsies may not be developed in full scale due to short reperfusion time.

#### KC depletion results in transient protection of livers from IRI.

**Figure 2:**
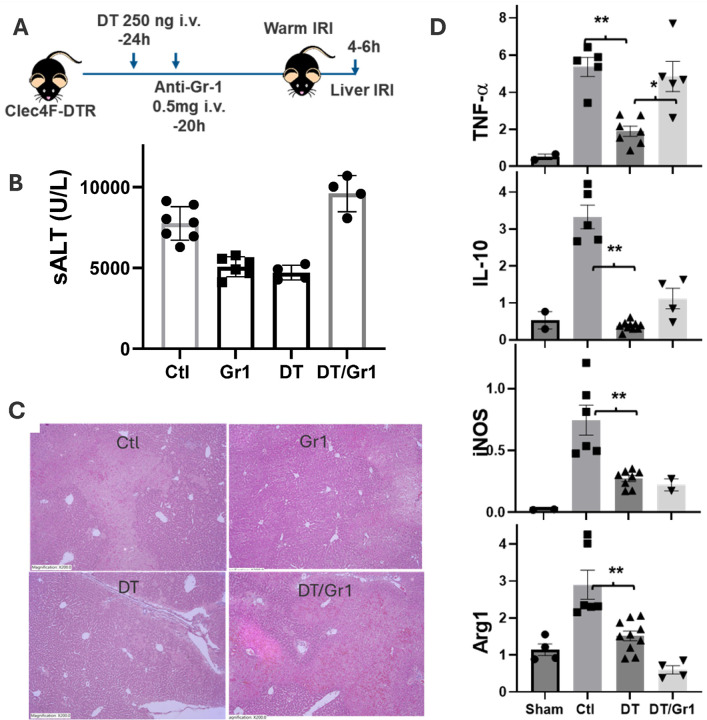
KC-specific depletion results in liver protection from IRI. (A) Groups of Clec4F-DTR mice were treated at 24h prior to the onset of liver ischemia with DT and/or anti-Gr-1Ab: Control, aGr-1, DT or DT/aGr-1. After 60m liver partial warm ischemia, IRI was evaluated at 6h post reperfusion. (B) Plot of average sALT levels in different exp. groups. (C) Representative liver histopathology (H/E staining, x200). (D) Plots of average ratios of target gene/HPRT of different exp. groups. Liver tissue RNA was isolated and analyzed for inflammatory gene expression by qRT-PCR. **p<0.01, *p<0.05.

To define the roles of KCs in liver IRI, we depleted KCs in Clec4F-DTR mice with a single dose of DT 24h prior to the onset of liver ischemia. The KC-specific depletion by DT in this model was confirmed by FACS analysis of MФs in livers and peritoneal cavity (Sup Fig. 1). Interestingly, these KC-depleted mice developed significantly lower levels of liver IRI (Fig. 2A), as documented by reduced sALT levels (measured at 6h post reperfusion), better preserved liver histological architectures and suppressed inflammatory gene induction in IR livers, as compared with controls (Fig. 2B, C, D).

We analyzed liver non-parenchymal cells (NPCs) by FACS at 24h post DT injection. The DT treatment resulted in a complete elimination of Clec4F^+^F4/80^+^CD11bl^ow^ KCs and replacement with F4/80^−^CD11b^+^Gr-1^+^(Ly6C^+^) infiltrating macrophages (Sup Fig. 4). To determine whether these Gr-1^+^ cells were responsible for the cytoprotective phenotype, we administered anti-Gr-1 Abs following DT injection (at −20h) in Clec4F-DTR mice (Fig. 2A). Indeed, the Ab treatment resulted in significant reduction of CD11b^+^Ly6C^+^(Gr-1^+^) cells in livers (Sup Fig. 4), and restored liver IRI in the KC-depleted mice (Fig. 2B), despite that the Ab by itself protected livers from IRI in KC-intact mice. There was concomitant upregulation of TNF-a and IL-10. These data indicate that the CD11b^+^ Gr-1^+^ infiltrating cells played immune suppressive roles in liver IRI.

To further test the immune regulatory properties of liver iMФs, we administered LPS intraperitoneally in groups of Clec4F-DTR mice treated with either PBS or DT at −24h. Peritoneal cells and livers were harvested from the same mice to determine tissue-specific responses in peritoneal cavity and liver. Indeed, the immune suppression was observed only in the livers of DT treated mice, as documented by the lower inflammatory gene induction, including TNF-a, IL-1b, IL-6 and IL-10 (Supplement Fig. 5). In the peritoneal cavities, there was no suppression of the induction of these genes in the DT vs. PBS treated mice.

### 2. The liver iMФs at 24h post DT injection exhibit immunosuppressive signatures.

**Figure 3:**
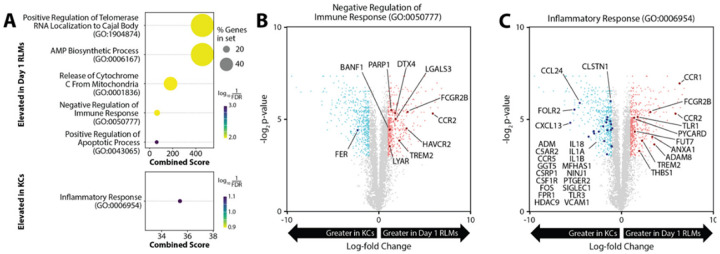
The differential gene expression analysis of 24h iMФs and KCs. (A) Dot plots depicting enrichment analyses for genes with elevated expression in 24h iMФs post-KC depletion (top) or Kupffer cells (KCs, bottom). (B-C) Volcano plots comparing differences in average gene expression between 24h iMФs and KCs. Colored points correspond to genes with log2-fold change greater than 1 and corrected t-test p-values below 0.05 between cell types. Red corresponds to genes with greater expression in 24h iMФs; blue corresponds to genes with greater expression in KCs. Bolded points with gene labels correspond to genes with significant differential expression that are part of the GO Biological Process terms “Negative Regulation of Immune Response” (B) and “Inflammatory Response” (C).

To analyze the protective mechanisms of these CD11b^+^Gr-1^+^ cells in 24h DT-treated livers, we analyzed the RNA-seq data set (GSE128657) of FACS-sorted liver MФs from Clec4F-DTR mice at different time points following DT injection deposited by Sakai M et al. (17). We compared average gene expression in KCs and iMФs 24h post-depletion by two-sample t-test. Differentially expressed genes (DEGs) were evaluated for concerted transcriptomic patterns via gene set enrichment analyses. Compared to KCs, 24h iMФs exhibited elevated expression of genes associated with negative regulation of immune responses (Fig. 3A). Likewise, KCs exhibited elevated expression of genes associated with inflammatory responses relative to the 24h iMФs. Further examination of differentials in gene expression found that immunoregulatory genes demonstrated near-universally greater expression in 24h iMФs relative to KCs (Fig. 3B). Inflammatory responses in KCs were driven primarily by genes associated with cytokine (IL1A, IL1B, IL18) and chemokine (CCL24, CCR5, CXCL13) responses (Fig. 3C). Comparatively, immunoregulatory responses in iMФs included elevated expression of Trem2, TIM-3 (HAVCR2), and Galectin-3, which have been shown to potently inhibit macrophage pro-inflammatory activation (27–29).

### 3. Trem2 is critical for the liver protective effect of Gr-1 monocytes

**Figure 4:**
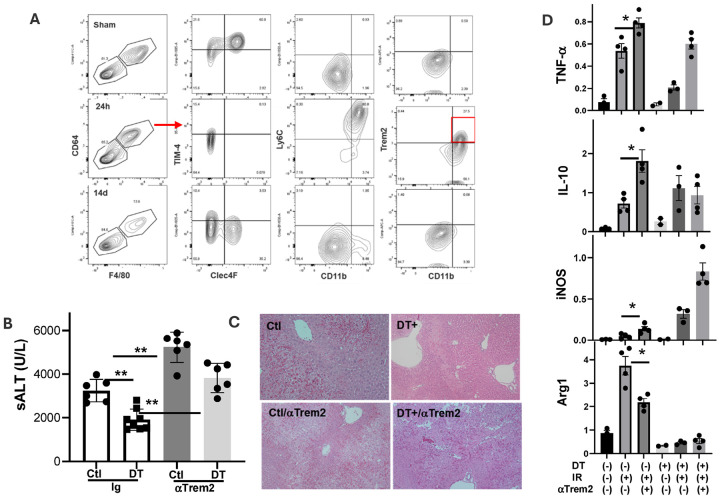
Trem2 is critical for the liver protective effect of Gr-1^+^ iMФs. (A) FACS analysis of liver myeloid cells in sham or DT-treated Clec4F-DTR mice at 24h or 14 days post DT injection. B) Plot of average sALT levels in different exp. groups. **p<0.01. (C) Representative liver histopathology pictures (H/E staining, x200) of different exp. groups. (D) Plots of average ratios of target gene/HPRT of different exp. groups. Liver tissue RNA was isolated and analyzed for inflammatory gene expression by qRT-PCR. *p<0.05.

To determine whether iMФs in KC-depleted livers expressed Trem2 at the protein level, we analyzed liver NPCs by FACS. F4/80 and CD64 first were used to identify MФs and non-MФs populations in CD45^+^ cells. KCs and non-KCs were differentiated based on TIM-4 and Clec2. Liver MФs are mostly TIM-4^+^Clec4F^+^ KCs in untreated mice but are TIM-4^−^ Clec4F^−^ iMФs in DT-treated ones. While KCs are CD11b^low^Ly6C^−^, these iMФs are CD11b^+^Ly6C^+^. Trem2 expression was indeed much higher in these iMФs vs. KCs (Fig. 4A).

To determine whether Trem2 was critical for the liver protective phenotype in KC-depleted mice, we tested the effect of anti-Trem2 blocking Abs (30, 31) in four groups of Clec4F-DTR mice: control, control/aTrem2, DT-treated, and DT-treated/aTrem2. DT was administered 24h and Abs were administered 1h, prior to the onset of liver ischemia. Interestingly, αTrem2 Ab increased liver IRI in both control and DT-treated mice, as compared with IgG controls (Fig. 4B,C). The liver injury increased most significantly in DT-treated mice, as the differences between control and DT groups were diminished by the αTrem2 Ab treatment. The inflammatory gene induction was also elevated by the Ab treatment in both control and DT-treated mice with distinctive patterns: the levels of IL-1b, IL-6 and IL-10 were increased most prominent in control mice, while those of TNF-a and iNOS were increased in DT-treated mice (Fig. 4D).

### 4. The liver protective phenotype was transient after KC depletion

**Figure 5:**
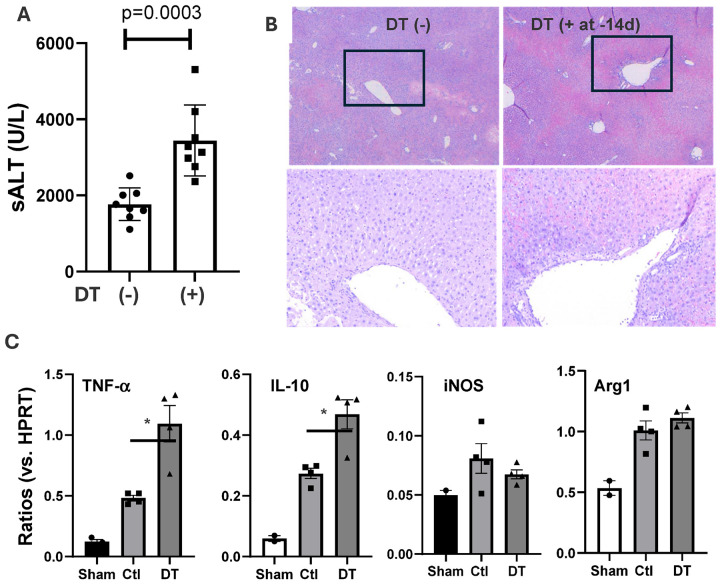
KC-depleted livers increase their susceptibility to IRI at day 14 post DT injection. Groups of Clec4F-DTR mice were untreated (Ctl) or treated with DT 14 day prior to the onset of liver ischemia. After 60m liver partial warm ischemia, IRI was evaluated at 6h post reperfusion. (A) Plot of average sALT levels of different exp. groups. (B) Representative liver histopathology (H/E staining, x50 and x200) pictures. (C) Plots of average ratios of target gene/HPRT of different exp. groups. Liver tissue RNA was isolated and analyzed for inflammatory gene expression by qRT-PCR.

The Gr-1^+^ iMФs in KC depleted livers of Clec4F-DTR mice evolved continuously. We isolated liver NPCs at 24h, 48h, 4d, 7d and 14d post DT injection and analyzed their phenotypes by FACS (Sup. Figure 6). Gr-1^+^CD11b^+^F4/80^low/−^ cells detected at 24h became CD11b^+^F4/80^+^ at 48h and Gr-1^−^CD11bl^ow^F4/80^+^ on day 4. To determine whether KC-depleted/ iMФ replaced livers remained protected after 24h, we performed liver IRI experiments at 48h, day 4, 7 and 14 post DT injection. Interestingly, the protective phenotype started to diminish at 48h, Liver IRI became similar between controls and DT treated ones at day 4 and 7 (Sup. Figure 6). Liver IRI was significantly increased at day 14 post DT treatment (Fig. 5), as documented by higher sALT levels, worse preserved liver histological architectures and higher inflammatory gene expressions in IR livers, vs. controls. We also analyzed Trem2 expressions at this time point. Indeed, Trem2^high^ MФs were not present. Majority of MФs were Cd11b^low^Ly6C^™^. Although Clec4F expression was partially restored, TIM-4 remained low/negative (Fig. 4A). These data indicate that iMФs in KC-depleted livers possess immune regulatory functions only transiently. They become pro-inflammatory as being continuously differentiated in livers.

### 5. Trem2 monocytes are present in liver CD11bLy6C infiltrates and blood.

**Figure 6:**
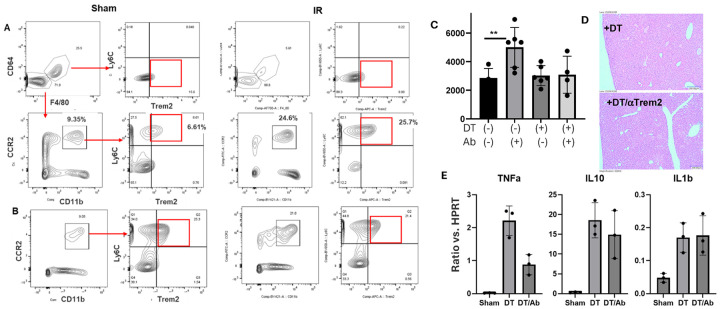
Trem2 expressions in liver monocytes and blood monocytes. (A) Liver NPCs were isolated from sham or IR livers of CCR2-GFP heterozygous mice. (B) Blood cells were also collected from the same mice. Cells were labeled with fluorochrome-labeled Abs and analyzed by FACS. CD45+ cells were gated for the analysis. Liver NPCs were first separated into macrophages and non-macrophages. Monocytes were identified in non-macrophages by CD11b and CCR2. Trem2 expressions were measured in macrophages and monocytes based on Ly6C levels. Blood monocytes were similarly measured for Trem2 expression. Representative plots were shown. N=3/group. (C). Average sALT levels of different exp. groups were plotted. **p<0.01. (D) Representative liver histopathology pictures (H/E staining, x200) were shown. Liver tissue RNA was isolated and analyzed for inflammatory gene expression by qRT-PCR. (E) Average ratios of target gene/HPRT of each exp. groups were plotted.

Since anti-Trem2 Abs augmented liver IRI in non-DT treated Clec4F-DTR mice, Trem2-mediated immune regulation is not limited to the iMФs in KC-depleted Clec4F-DTR mice. To determine which myeloid cells express Trem2 in livers, we isolated NPCs from either sham or IR livers of CCR2-GFP heterozygous mice. In non-macrophage cells (CD45^+^CD64^−^F4/80^−^), we found that CD11b^+^CCR2^+^Ly6C^+^ monocytes expressed the highest average level of Trem2 (Fig. 6A), as compared with macrophages (CD45^+^CD64^+^F4/80^+^). As Ly6C + monocytes represent recent infiltrates from blood. We also measured Trem2 levels in blood monocytes (Fig. 6B). Indeed, CD11b + CCR2 + Ly6C + blood monocytes also expressed Trem2, similar to their liver infiltrating counterparts. Liver IR resulted in significant increases of liver Ly6C + infiltrating monocytes, as well as blood CCR2 + monocytes, with the Trem2^high^ phenotype. Meanwhile, resident macrophages were depleted drastically in IR livers. Thus, These Trem2^high^ monocytes might represent cytoprotective cells in the activation phase of liver IRI.

To determine whether CD11b + CCR2 + Ly6C + monocytes mediated the immune regulatory effect of Trem2 in liver IRI, we depleted CD11b cells in CD11b-DTR mice and tested the effect of anti-Trem2 Ab. Indeed, the Abs were no longer able to augment liver IRI in the absence of CD11b cells (Fig. 6C, D, E), as documented by sALT levels, histopathological pictures and inflammatory gene expressions.

### 6. Broad inhibition of CD11b + CCR2 + monocytes does not change the severity of liver IRI.

**Figure 7:**
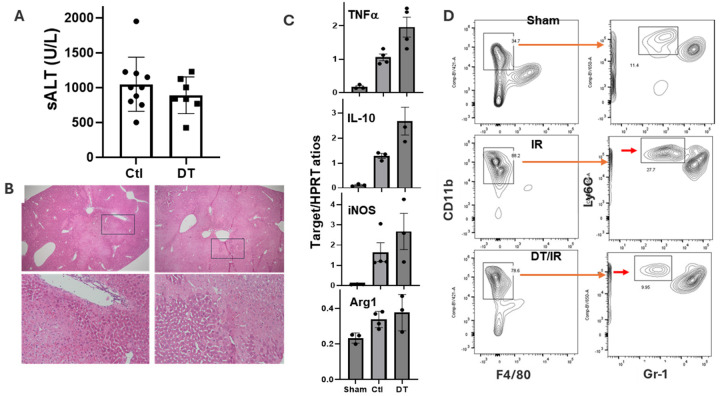
CD11b depletion reduces Gr-1 monocyte infiltration in the acute phase of liver IRI, but not the severity of hepatocellular damages. Groups of CD11b-DTR mice were either treated with PBS or DT at 24h prior to the onset of liver ischemia. Liver IRI was induced by 60m ischemia and followed by 6h reperfusion. (A) Plot of average sALT levels in different exp. groups. (B) Representative liver histopathology (H/E staining, x50 and x200) pictures. (C) Average ratios of target gene/HPRT of each exp. groups were plotted. Liver tissue RNA was isolated and analyzed for inflammatory gene expression by qRT-PCR. (D) FACS analysis of liver myeloid cell infiltration. F4/80-CD11b+ cells were gated and further analyzed based on Gr-1 and Ly6C. Red arrows indicate Gr-1 monocytes. Representative plots were shown (n=3/group).

As a comparison, we also studied CD11b-DTR and CCR2 deficient mice to determine roles of BM-derived iMФs in our model. CD11b depletion by a single dose of DT 24h prior to the onset of liver ischemia did not affect the severity of liver IRI, as shown by sALT levels, histopathological pictures and inflammatory gene expressions (Fig. 7A, B, C). Similarly, CCR2 deficient (CCR2-GFP homozygous) mice developed the same levels of liver IRI as their controls (CCR2 heterozygous), indicating that inhibition of CCR2 + myeloid cell infiltration does not impact the acute phase of liver IRI (Sup Fig. 7).

FACS analysis of liver myeloid cells in CD11b-DTR mice showed that DT treatment resulted in a significant reduction of CD11b^+^Ly6C^+^ monocytes without affecting neutrophil infiltrates (CD11b^+^Ly6C^+^ Gr-1^high^) in IR livers (Fig. 7D). CCR2 deficiency did not affect CD11b^+^Ly6G^+^Ly6C^low^ neutrophils but inhibited the infiltration of all CCR2^+^CD11b^+^Ly6C^+^Ly6G^−^ monocytes in IR livers (Sup Fig. 8D). These data demonstrated that broad suppression of monocytes or their infiltration does not impact the severity of acute liver IRI.

### 7. KCs promote liver IRI via IL-1a and IL-1b.

DEG analysis revealed that KC inflammatory response was driven primarily by IL-1 family genes (Fig. 3). In KC-depleted/iMФ repopulated livers, we found that IL-1a/b gene expressions were drastically decreased in both sham and IR conditions, indicating that KCs were the main cellular source of IL-1 genes. To confirm KC expression of IL-1a/b at the protein level, we performed intracellular staining (precursors). KCs isolated from sham mice expressed IL-1a but not -b. Liver IR increased IL-1a and induced IL-1b expressions (Sup. Figure 8). Interestingly, IL-1a was only detected in KCs while IL-1b was expressed in all myeloid cells. To test whether IL-1 genes were critical for KC functions in liver IRI, we compared the effect of anti-IL-1a Abs in four groups of Clec4F-DTR mice, control/aIL-1a, DT-treated, and DT-treated/aIL-1a. Indeed, IL-1a neutralization reduced liver IRI in control (KC-intact), but not in KC-depleted (DT-treated) mice, as documented by sALT levels and histopathological pictures (Fig. 7A, B). The inflammatory gene induction, including TNF-a, and IL-10, Arg1, IL-1a and b were suppressed by the Ab treatment in control, but not DT-treated, mice (Fig. 7C). Similar results were observed with anti-IL-1b neutralizing Abs (Sup. Figure 9). These results indicate that KCs promote liver IRI via IL-1a/b.

## Discussions

Taking advantage of KC lineage tracing mice (Clec4F-tdTomato) and markers such as Clec2, TIM-4 (16, 17, 32), we have found that liver IR results in significant depletion of KCs, simultaneously with infiltrations of neutrophils and monocytes in the acute phase (6h post reperfusion) of liver IRI. When we deplete KCs with DT prior to the onset of liver ischemia, iMФs repopulate the liver and transiently protect from IRI. The cytoprotective iMФs are Gr-1^+^ with immune regulatory gene expression signature and exert their function in Trem-2-dependent manner. The Trem2^high^ monocytes are also present in KC-intact livers and blood and protect livers from IRI. Based on the DEG analysis, we confirm that KCs express IL-1a and b. Ab-mediated blockade of these cytokines mitigates liver IRI in KC-intact, but not -depleted, mice. our study is the first to specify KC changes and functions in liver IRI. The results challenge the prevailing paradigm in the field and illustrate a unique mechanism of inflammatory activation in liver IRI: KCs promote, while iMФs restrain, liver inflammation/injury by IL-1a/b and Trem2 signaling pathways, respectively.

Roles of KCs in liver IRI have been controversial. Early studies using clodronate liposomes (CL)-mediated depletion approach have shown both aggravation of liver IRI with enhancement of liver inflammatory immune response (33, 34), as well as protection of livers from IRI (35, 36). The expression of heme oxygenase-1 (HO-1) and production of IL-10 have been proposed as mechanisms of KC-mediated liver protection (33, 34). Our own studies using either CLs or anti-TIM-4 Abs at 48h prior to the onset of liver ischemia all show increased liver IRI in these putative “KC”-depleted mice (14, 15). However, CLs are taken up by all phagocytes, including monocytes, without differentiating their origin (tissue resident or bone marrow derived). Even neutrophils are shown recently to ingest the liposomes, which impair their functions and result in the anti-inflammatory effects of CLs (37). Although TIM-4 can differentiate tissue resident vs. bone marrow derived macrophages, whether the anti-TIM-4 Ab really depletes the cells or masks TIM-4 molecules remain controversial (our own data). Additionally, TIM-4 is expressed by other cells, including T cells, NK cells, which are alsp involved in liver IRI. Thus, Clec4F-DTR mice is the most specific tool available nowadays to dissect roles of KCs in liver diseases. Results of the KC-depletion experiment are “unexpected” in several aspects. First, the depletion of tissue resident MФs does not result in an “empty” organ, but rather the replacement with iMФs. The functional differences of these iMФs vs. the original resident MФs determines the outcome of the depletion effect. Second, iMФs are initially cytoprotective, despite the Gr-1^+^/Ly6C^+^ pro-inflammatory phenotype. Third, iMФs evolve continuously in livers not only phenotypically but also functionally, leading to changes in liver susceptibility to IRI. When tested at 14 days post DT injection, they become pro-inflammatory, as compared with embryonically derived KCs (TIM-4+), resulting in higher levels of liver IRI.

Clearly, more questions are raised about how KCs function in liver IRI. As they were lost in the disease process, the production of IL-1 gene products must be associated with cell death. Interestingly, it has been shown that IL-1a is released from necrotic cells (38), while IL-1b is a product of inflammasome activation and released from pyroptotic cells (39, 40). Both IL-1 gene products are synthesized in cells as inactive precursors and required post-translational cleavage by protease, including caspases, to generate active cytokines. Thus, our future study will focus on whether KCs produce active IL-1a/b while undergoing necroptosis or pyroptosis. Our previous study have demonstrated that KC necroptosis is critical for their function in liver IRI that Nec-1s treatment was able to reduce the loss of KCs and protect livers in KC-repleted, but not -depleted (by clodronate liposomes) mice (14).

The immune regulatory Gr-1^+^ iMФs express high levels of Trem2, which is the canonical marker of a new type of liver MФs, lipid-associated macrophages (LAM). LAMs have been implicated in liver cirrhosis, as pro-fibrogenic scar-associated MФs; in cancer, as immunosuppressive TAMs, and most recently in acute liver injury models, as tissue reparative MФs (8, 41, 42). It has been shown that LAMs can derive from either KCs or BM-derived iMФs (42). Trem2 binds to multiple ligands. As a lipid binding receptor, it can initiate macrophage efferocytosis and phagocytosis. Trem2 potently modulates TLR-mediated macrophage activation primarily by DAP12-mediated inhibition of Erk activation and recruitment of SHIP1 (28, 31, 41). In liver IRI, the global deficiency of Trem2 or DAP12 increased the severities of acute hepatocellular injuries mediated in part by DCs (43). A more recent study using myeloid Trem2 deficient mice, however, showed a protective phenotype in the acute phase but delayed inflammation resolution later in the disease process (44). Our experiment with an anti-Trem2 blocking Ab revealed its broad immune regulatory roles in liver IRI in both KC-intact and -depleted mice. We have detected CD11b^+^CCR2^+^Trem2^high^ monocytes in both liver NPCs and blood, which were increased by liver IR. Depletion of CD11b cells abrogated the anti-Trem2 effect in CD11b-DTR mice, suggesting that Trem2 regulates infiltrating monocytes in liver IRI. It remains to be determined whether anti-Trem2 also targeted LAMs or DCs in our model. However, these resident myeloid cells are substantially depleted by liver IR and quantitatively much smaller than infiltrating monocytes.

Liver IRI is associated with rapid and extensive infiltration of neutrophils and monocyte/macrophages, which are thought to play important roles in the activation of liver inflammatory response. However, few studies differentiated specifically iMФs from KCs in liver IRI models (33, 45). In fact, the first report using CD11b-DTR mice showed no effect of CD11b depletion on liver IRI (33). CCR2 is critical for the infiltration of inflammatory monocyte/macrophages in diseased livers (12, 46–48). CCR2 deficient mice developed reduced liver IRI (36). In our study, we observed minimal impact of DT treatment in CD11b-DTR mice and CCR2 deficiency on liver IRI. FACS analysis confirmed that only (CCR2+) CD11b + Ly6C + monocytes, but not neutrophils, were inhibited in liver infiltrations in these mice. Thus, disruption of total monocytes infiltration does not alter the severity of liver IRI. However, selective blocking of Trem2 in Ly6C^+^CD11b^+^ monocytes protect livers from IRI. These results indicate functional heterogeneity in infiltrating monocytes, which may consist of both pro- and anti-inflammatory types.

In summary, our study presents the first evidence that liver-resident KCs play a pivotal role in initiating inflammatory responses during liver IRI through IL-1α/b production. The discovery of a distinct population of immune regulatory monocytes/iMΦs—characterized by a (Gr-1^+^) Ly6C^+^CD11b^+^Trem2^high^ phenotype—further underscores the complexity of liver macrophage/monocyte heterogeneity. A comprehensive understanding of these diverse macrophage subsets, both in phenotype and function, is essential for the development of targeted cell-based therapies aimed at mitigating IRI and enhancing graft tolerance.

## Supplementary Material

Supplementary Files

This is a list of supplementary files associated with this preprint. Click to download.

- Sup.docx

## Figures and Tables

**Figure 8:**
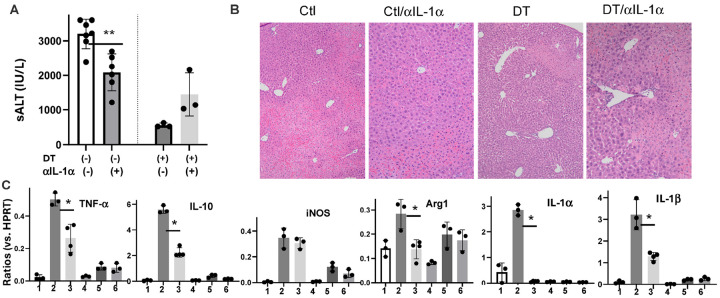
KCs promotes liver IRI via IL-1a. A) Average sALT levels of indicated exp. groups. **p<0.01. (B) Representative liver histopathology pictures (H/E staining, x200) of indicated exp. groups. (C) Average ratios of target gene/HPRT of different exp. groups: 1. Sham, 2. IR/Ctl IgG, 3. IR/anti-IL-1a; 4. DT/Sham, 5. DT/IR/Ctl IgG, 6. DT/IR/anti-IL-1a. *p<0.05.

**Table 1:** Primers for qRT-PCR

Primer	Forward Sequence	Reverse Sequence
TNF-a	ACTGAACTTCGGGGTGATCG	CTACAGGCTTGTCACTCGAA
IL-10	CCAGCTGGACAACATACTGC	CCAGCTGGACAACATACTGC
iNOS	GGGACTGAGCTGTTAGAGACAC	GCACAAGGGGTTTTCTTCACG
IL-1a	ACGTCAAGCAACGGGAAGAT	AAGGTGCTGATCTGGGTTGG
IL-1b	TGCCACCTTTTGACAGTGATG	AAGGTCCACGGGAAAGACAC
Arg1	GTGGGGAAAGCCAATGAAG	GCTTCCAACTGCCAGACTGT

## Abbreviations

Abs: antibodies
BM: bone marrow
DTR: diphtheria toxin receptor
FACS: fluorescence-activated cell sorting
GFP: green fluorescent protein
iMФ: infiltrating macrophages
KCs: Kupffer cells
KO: knock-out
IRI: ischemia-reperfusion injury
NPCs: non-parenchymal cells
sALT: serum alanine aminotransferase
